# Mapping tissue water *T*_1_ in the liver using the MOLLI *T*_1_ method in the presence of fat, iron and *B*_0_ inhomogeneity

**DOI:** 10.1002/nbm.4030

**Published:** 2018-11-21

**Authors:** Ferenc E. Mozes, Elizabeth M. Tunnicliffe, Ahmad Moolla, Thomas Marjot, Christina K. Levick, Michael Pavlides, Matthew D. Robson

**Affiliations:** ^1^ The University of Oxford Centre for Clinical Magnetic Resonance Research (OCMR), University of Oxford, John Radcliffe Hospital Oxford UK; ^2^ Oxford Centre for Diabetes, Endocrinology and Metabolism (OCDEM) University of Oxford, Churchill Hospital Oxford UK; ^3^ Translational Gastroenterology Unit University of Oxford, John Radcliffe Hospital Oxford UK; ^4^ Oxford NIHR Biomedical Research Centre Oxford UK

**Keywords:** fat, iron, MOLLI, NAFLD, off‐resonance, shMOLLI

## Abstract

Modified Look‐Locker inversion recovery (MOLLI) *T*_1_ mapping sequences can be useful in cardiac and liver tissue characterization, but determining underlying water *T*_1_ is confounded by iron, fat and frequency offsets. This article proposes an algorithm that provides an independent water MOLLI *T*_1_ (referred to as on‐resonance water *T*_1_) that would have been measured if a subject had no fat and normal iron, and imaging had been done on resonance. Fifteen NiCl_2_‐doped agar phantoms with different peanut oil concentrations and 30 adults with various liver diseases, nineteen (63.3%) with liver steatosis, were scanned at 3 T using the shortened MOLLI (shMOLLI) *T*_1_ mapping, multiple‐echo spoiled gradient‐recalled echo and ^1^H MR spectroscopy sequences. An algorithm based on Bloch equations was built in MATLAB, and water shMOLLI *T*_1_ values of both phantoms and human participants were determined. The quality of the algorithm's result was assessed by Pearson's correlation coefficient between shMOLLI *T*_1_ values and spectroscopically determined *T*_1_ values of the water, and by linear regression analysis. Correlation between shMOLLI and spectroscopy‐based *T*_1_ values increased, from *r* = 0.910 (*P* < 0.001) to *r* = 0.998 (*P* < 0.001) in phantoms and from *r* = 0.493 (for iron‐only correction; *P* = 0.005) to *r* = 0.771 (for iron, fat and off‐resonance correction; *P* < 0.001) in patients. Linear regression analysis revealed that the determined water shMOLLI *T*_1_ values in patients were independent of fat and iron. It can be concluded that determination of on‐resonance water (sh)MOLLI *T*_1_ independent of fat, iron and macroscopic field inhomogeneities was possible in phantoms and human subjects.

**Abbreviations used**
AMARES: advanced method for accurate, robust and efficient spectral fitting
bSSFP: balanced steady‐state free precession
ECF: extracellular fluid volume fraction
GRE: gradient recalled echo
HIC: hepatic iron concentration
MCSE: multiple contrast spin echo
MOLLI: modified Look‐Locker inversion recovery
NAFLD: non‐alcoholic fatty liver disease
PDFF: proton density fat fraction
shMOLLI: shortened MOLLI
STEAM: stimulated echo acquisition mode
TI: inversion time
TM: mixing time

## 1 INTRODUCTION

Quantifying the longitudinal relaxation time constant (*T*_1_) in clinical practice is often performed using the modified Look‐Locker inversion recovery (MOLLI) method^1^ or its variants,^2^, ^3^ due to their speed, precision, availability on clinical scanners and good reproducibility.^4^ This method has proved to be diagnostically useful both in cardiovascular MRI^5^ and in liver MRI, correlating with fibrosis and inflammation^6^ without the risks of biopsy.^7^ Liver *T*_1_ maps were also found to predict clinical outcomes in patients with chronic liver disease.^8^

However, *T*_1_ measured with MOLLI methods is sensitive not only to fibro‐inflammatory disease but also to a number of confounding factors that change the *T*_1_ or its measurement, either by altering the microscopic magnetic environment of the imaged tissue or by means of partial volume effects.

MOLLI and its variants generally employ a balanced steady‐state free precession (bSSFP) readout.^1^ The signal produced by bSSFP is dependent on the *T*_1_ and *T*_2_ of imaged proton species, flip angle and off‐resonance frequency.^9^ The frequency dependence, in particular, can result in complex behaviour of the bSSFP signal during *T*_1_ recovery when the imaged voxels contain both fat and water.^10^, ^11^, ^12^ With typical imaging parameters and clinically relevant fat fraction, this leads to an overestimation of the liver water MOLLI *T*_1_, at odds with the decrease expected in a simple partial volume model. This counterintuitive increase in MOLLI *T*_1_ with fat is in addition to the effect of iron deposits that reduce *T*_1_ and *T*_2_.^13^, ^14^ Magnetization transfer,^15^ heart rate,^2^ repetition time of the bSSFP readout^10^ and *B*_0_ inhomogeneity^10^ also influence MOLLI *T*_1_ values.

Removing the effect of iron on MOLLI *T*_1_ has previously been demonstrated,^16^ but to best characterize fibro‐inflammatory disease off‐resonance effects need to be removed, both *B*_0_ related and chemical shift related, to produce an on‐resonance water MOLLI *T*_1_. A disease where this might prove to be important is non‐alcoholic fatty liver disease (NAFLD), a condition characterized by hepatic lipid accumulation.^17^ NAFLD has a worldwide prevalence of 20–30%,^18^, ^19^ and encompasses a range of pathology from non‐alcoholic steatosis (simple fat accumulation) to non‐alcoholic steatohepatitis (fat accumulation associated with liver inflammation) to cirrhosis. In some cases, hepatocellular carcinoma may develop.^20^, ^21^ Since both the presence of fat and fibro‐inflammation cause MOLLI *T*_1_ values to increase at 3 T when using TR ≈ 2.3 ms, differentiating these two processes may provide additional insight into the disease and its progression.

The present study evaluates whether the confounding effect of lipids and off‐resonance frequency can be, in addition to the previous correction for the effect of iron, taken into account to provide a MOLLI *T*_1_ independent of the influence of these factors. Therefore, the target is a water MOLLI *T*_1_ value that would have been measured if a subject had no fat and normal iron, and imaging had been done on resonance.

## 2 METHODS

### 2.1 Phantom experiments

#### 2.1.1 Construction

Fifteen agar‐based phantoms were built with varying proportions of fat and water. Phantoms were stored in 30 mL plastic sample containers (King Scientific, Huddersfield, UK) and were built in three batches, using a recipe similar to that described by Hines et al.^22^ To vary the *T*_1_ of water in each of the batches, NiCl_2_ (Sigma‐Aldrich, St. Louis, MO, USA) solutions with concentrations of 0.45 mM, 0.73 mM and 1.61 mM were used. Next, 2%/weight agar (Sigma‐Aldrich), 0.01%/weight sodium benzoate (Sigma‐Aldrich), 43 mM sodium chloride (Sigma‐Aldrich) and 43 mM sodium dodecyl sulfate (Sigma‐Aldrich) were added. Each of the three solutions was boiled until it became transparent and viscous. Five sample containers were prepared for each batch, having 0%, 5%, 10%, 20% and 30%/vol. of peanut oil (Tesco, Welwyn Garden City, UK). Each container was filled to 30 mL with water‐agar solution. Each sample container was further homogenized for 5 min using an ultrasonic homogenizer (Eumax UD200SH, Hong Kong). The sodium dodecyl sulfate in the solution ensured that water and fat formed a homogenous emulsion. A 16th sample container was filled with 100% peanut oil. Peanut oil was chosen due to its ^1^H spectrum resembling the ^1^H spectrum of human subcutaneous fat.^23^

#### 2.1.2 Imaging

Phantoms were scanned using a Siemens Tim Trio 3 T imager (Siemens Healthineers, Erlangen, Germany) equipped with a six‐channel body matrix coil (Siemens Healthineers) and a spine array coil (Siemens Healthineers) with 24 channels, of which nine were used. A set of multiple‐echo spoiled gradient recalled echo (GRE) images was collected to compute a map of the main magnetic field variation (*γ* Δ*B*_0_). Parameters of the 2D multiple‐echo GRE sequence were flip angle (FA) = 6°, TR/TE = 22.2/1.25, 2.46, 3.69, 4.92, 6.15, 7.38, 8.61, 9.84 ms, field of view (FOV) 302 × 245 mm^2^, acquisition matrix 128 × 104, phase encoding direction anterior–posterior, BW = 1180 Hz/px, bipolar gradient readout scheme and slice thickness 7 mm. The map of *γ* Δ*B*_0_ field variation was determined from a *T*_2_*‐IDEAL fat‐water separation method with field map estimation using a graph‐cut algorithm.^24^, ^25^

*T*_2_ values of the fat‐free phantoms were determined using a multiple contrast spin echo (MCSE) experiment to quantify the effect of the agar and NiCl_2_ on transverse relaxation. The following sequence parameters were used: TR/TE = 9000/15–480 ms in steps of 15 ms, FOV 270 × 270 mm^2^, matrix 128 × 110, slice thickness 8 mm. Images with different contrasts were fitted to a mono‐exponential decay model. A MOLLI variant, shortened MOLLI (shMOLLI),^2^ was used to acquire a *T*_1_ map by collecting images at seven inversion times, over nine simulated heart beats with three inversions, using the 5(1)1(1)1 scheme, i.e. the first inversion pulse was followed by five readouts and a pause with a duration of one RR interval, the second inversion pulse was followed by one readout and a pause with a duration of one RR interval and the third inversion pulse was followed by one readout. ShMOLLI parameters followed a standardized protocol (Siemens WIP 561a, Erlangen, Germany): readout FA = 35°, TR/TE = 2.52/1.05 ms, FOV 290 × 343 mm^2^, matrix 192 × 182, phase encoding direction anterior–posterior, BW = 898 Hz/px, slice thickness 8 mm, 28 lines before the central line, 82 total *k*‐space lines, shortest inversion time (TI) = 110 ms, inversion time increment 80 ms, generalized autocalibrating partially parallel acquisition (GRAPPA)^26^ acceleration factor 2 with 24 reference lines, partial Fourier factor 6/8 in the phase encoding direction and simulated heart rate of 60 beats/min. The approach to steady state was accelerated using five linearly increasing start‐up angle (LISA) pulses^27^, ^28^ and one half‐angle ramp down pulse. *T*_1_ values were obtained by applying the shMOLLI conditional fitting algorithm.^2^ All images were acquired in a coronal slice.

#### 2.1.3 Spectroscopy

A set of ^1^H spectra with multiple repetition times (TR) and echo times (TE) was collected using a multiple‐TR, multiple‐TE stimulated echo acquisition mode (STEAM) single‐voxel MRS sequence^29^ to characterize the *T*_1_ and *T*_2_ values of six fat peaks in the pure peanut oil phantom. Relaxation times of the pure peanut oil phantom were repeated at 20°C and 37°C. The multiple‐TR, multiple‐TE STEAM sequence followed the approach described by Hamilton et al.^29^: no water suppression, voxel size 14 × 14 × 24 mm^3^, mixing time (TM) = 9 ms, 24 spectra collected with TR varying between 150 and 2000 ms (150, 175, 200, 225, 250, 275, 300, 325, 350, 400, 450, 500, 600, 700, 800, 900, 1000, 1250, 1500, 2000 ms) and TE fixed at 15 ms, then eight spectra collected with a fixed TR of 1000 ms and TE changing from 20 to 110 ms (20, 25, 30, 35, 50, 70, 90, 110 ms). A version of the sequence with multiple TR but fixed TE = 15 ms was used to quantify individual *T*_1_ values of water and fat peaks in the mixed water and oil phantoms. Proton density fat fractions (PDFFs) were also determined from ^1^H spectroscopy data. The obtained spectra were fitted using the MATLAB‐adapted version of the advanced method for accurate, robust and efficient spectral fitting (AMARES) algorithm,^30^, ^31^ and the resulting amplitudes for each peak were fitted to Equation 1, where *S*_0*i*_ is a scaling factor proportional to proton density, TE is the echo time, *τ* is the time from the third 90° pulse of the STEAM sequence to the end of the TR interval, i.e. the time during which the longitudinal magnetization recovery occurs, and *i* is an index for each spectral peak. Table 1 shows the prior knowledge used for the AMARES fitting.

(1) $$S_{i}=S_{0i}\left(1-\exp\left(-\frac{\tau }{T_{1i}}\right)\right)\exp\left(-\frac{\mathrm{TE}}{T_{2i}}\right).$$

**Table 1:** Prior knowledge of six fat peaks used to fit peanut oil spectra

**Peak number**	**Chemical shift [ppm]**	**Line shape**	**Line width [Hz]**	**Peak name**
1	0.90	Lorentzian	20	Methyl
2	1.30		40	Methylene
3	2.10		40	α‐carboxyl and α‐olefinic
4	2.76		20	Diacyl
5	4.30		40	Glycerol
6	5.30		30	Olefinic

When analysing multiple‐TR only experiments, the *T*_2*i*_ were fixed to be those determined in the pure peanut oil phantom and the 0% fat phantom. PDFF was defined as the ratio of the sum of observed fat proton densities and the sum of proton densities of water and observed fat peaks, as defined by Equation (2). Here $S_{0f_{i}}$ denotes the proton density of the *i*th fat peak and *S*_0w_ is the proton density of water. These values were determined from fitting spectroscopy data to Equation 1.

(2) $$\text{PDFF}\;\left[\%\right]=\frac{\sum_{i}S_{0f_{i}}}{S_{0w}+\sum_{i}S_{0f_{i}}}\times 100.$$

#### 2.1.4 Simulation

A Bloch equation simulation of the shMOLLI sequence was built in MATLAB (MathWorks, Natick, MA, USA). The simulation used the same parameters for the shMOLLI protocol as were used during the imaging experiments. The measured signal was determined as the average signal over 59 phase encode lines centred at *k* = 29. A hyperbolic secant 1 inversion pulse of 10.24 ms duration was used for inversion with time‐bandwidth product *R* = 5.48, peak *B*_1_ = 750 Hz and *β* = 3.45. The evolution of magnetization over time was simulated using Brian Hargreaves' Bloch equation simulator (http://mrsrl.stanford.edu/~brian/blochsim/).

Fat was characterized using the same six spectral peaks as evaluated by MRS. The chemical shift offset relative to water, relative amplitude and *T*_1_ and *T*_2_ relaxation times for each simulated component were determined from the multiple‐TR, multiple‐TE STEAM spectra of the peanut oil phantom.

As an initial test of the phantom model, three simulations of the water signal were carried out corresponding to the three NiCl_2_ concentrations, using the water *T*_2_ values measured using the MCSE experiment and average STEAM water *T*_1_ values for the given NiCl_2_ concentrations. The water and fat bSSFP signals were then combined according to the principle of partial volumes to reflect the PDFF determined from the multiple‐TR, multiple‐TE STEAM spectroscopy experiment for each fat‐water phantom and input to the shMOLLI *T*_1_ fitting algorithm. These simulated shMOLLI *T*_1_ values were then compared with the measured shMOLLI *T*_1_ as described in the statistics section.

#### 2.1.5 Determining the water shMOLLI *T*_1_

To determine the shMOLLI *T*_1_ of the water component of the phantoms, the simulations above were repeated with water *T*_1_ varied over the 500–1600 ms range in steps of 1 ms, and the water and fat signals were again combined according to the measured PDFF. The simulated signal that satisfied the maximization problem described by Equation 3 was selected as the closest to the measured signal, where *s* represents simulated bSSFP signal vectors, *s*_meas_ is the measured bSSFP signal vector, *θ* is the angle between the simulated and measured signal,vectors and the numerator of the fraction represents the absolute value of the dot product of the two vectors while the denominator represents the product of the lengths of the two vectors).

(3) $$s=\underset{s\in {\mathbb{C}}^{7}}{\text{argmax}}\cos\theta_{s-s_{\text{meas}}}=\underset{s\in {\mathbb{C}}^{7}}{\text{argmax}}\frac{|s\cdot s_{\text{meas}}|}{\left\|s\right\|\cdot \left\|s_{\text{meas}}\right\|}.$$

Once the maximization was complete, an additional Bloch simulation was carried out to obtain the water‐only signal that would have been generated by the MOLLI sequence on resonance. This signal was then fitted to Equation 4 to determine the apparent *T*_1_ (*T*_1_*). ShMOLLI *T*_1_ was obtained using the imperfect^32^ Look‐Locker correction^1^, ^32^ as described by Equation 5.

(4) $$S_{\mathrm{sim}}=A-B\exp\left(-\frac{TI}{T_{1}^{*}}\right)$$

(5) $$T_{1}=T_{1}^{*}\left(\frac{B}{A}-1\right).$$

The *T*_1_ obtained in the last step was considered to be the water shMOLLI *T*_1_ value. This process was repeated for each of the phantom vials.

### 2.2 Patient study

#### 2.2.1 Patient population

A total of 30 patients (18 female, mean age: 53 ± 8.3 years) with normal livers (*N* = 11), alcoholic liver disease (*N* = 1) and NAFLD (*N* = 18) were included. Participants were recruited from a hepatology clinic. Inclusion criteria were: adults aged between 18 and 75 years old, BMI between 25 and 50 kg/m^2^, 1.5 ≤ ALT <10 ULN, blood pressure < 160/100 mm Hg and primary diagnosis of NAFLD. Exclusion criteria were: diagnosis of diabetes, blood haemoglobin <120 mg/dL, haemorrhagic disorders, anticoagulant treatment or presence of any contraindications to an MRI examination.

The study conformed to the ethical guidelines of the 1975 Declaration of Helsinki, and was approved by the institutional research departments and the National Research Ethics Service. All patients gave written informed consent.

#### 2.2.2 Imaging

Patients were scanned using the same scanner and coils as described in the phantom work and using the same imaging and spectroscopic sequences. Six or nine channels of the spine array coil were used, depending on patient liver size.

*T*_2_* values were obtained using a *T*_2_*‐IDEAL fat‐water separation method with field map estimation using a graph‐cut algorithm and a signal model described by Hernando et al.^25^ (Equation 6).

(6) $$S\left(\rho_{w},\rho_{f},T_{2}^{*},{\mathrm{TE}}_{n}\right)=e^{-\frac{{TE}_{n}}{T_{2}^{*}}}\left(\rho_{w}+\rho_{f}\sum_{k=1}^{P}\alpha_{k}\;e^{2\mathrm{\pi i}f_{k}{TE}_{n}}\right)e^{-2\mathrm{\pi i\psi}{\mathrm{TE}}_{n}}.$$

Here, *ρ*_w_ and *ρ*_f_ stand for the relative proton densities of water and fat, *ψ* represents field inhomogeneities, *T*_2_* is common for fat and water, TE_*n*_ represents the *n*th echo time, *α*_*k*_ is the relative amplitude of the *k*th fat peak, *f*_*k*_ is the chemical shift offset of the *k*th fat peak and *p* is the total number of fat peaks (*p* = 6 in our model). Equation 7 was then applied to obtain hepatic iron concentration (HIC) values in milligrams per gram dry weight of hepatic tissue.^33^, ^34^

(7) $$\mathrm{HIC}\;\left[\mathrm{mg}/g\;d.w.\right]=\frac{\left(\frac{1}{T_{2}^{*}}+11\right)}{2}\times 0.0254+0.202.$$

Hepatic shMOLLI *T*_1_ maps had TR = 2.45 ms, bSSFP TE varied between 1.02 and 1.05 ms, FOV was (270–300) × (360–400) mm^2^, there were 24 *k*‐space lines before the central line and 66 total *k*‐space lines, the shortest inversion time was either 100 ms or 110 ms and the inversion time increment 80 ms.

Additionally, a single‐voxel long‐TR STEAM ^1^H spectroscopy sequence^35^ was also run with and without water suppression. These spectra were used to quantify PDFF. Sequence parameters were the following: TR was cardiac gated and at least 2 s for suppressed spectra and at least 4 s for non‐suppressed spectra, TE = 10 ms and TM = 7 ms. Voxel size was 20 × 20 × 20 mm^3^ for both spectroscopy experiments. Water suppressed and non‐water‐suppressed spectra were processed using AMARES and combined with a specialized MATLAB script.^35^ When fat peaks were not visible, their amplitudes were determined using the relative amplitudes reported by Hamilton et al.^36^ PDFF was determined as the ratio of the sum of areas of fat peaks and the sum of water and fat peak areas, and was *T*_2_ corrected using Equation 8.

(8) $$\text{PDFF}\;\left[\%\right]=\frac{\sum_{i}F_{i}}{W\;e^{\mathrm{TE}\;R_{2}}+\sum_{i}F_{i}}\times 100$$

where *F*_*i*_ are the fitted amplitudes of fat peaks, *W* is the fitted amplitude of the water peak, TE is the spectroscopic echo time and *R*_2_ is the transverse relaxation rate of the water in the liver in 1/s, given by Equation 9,^33^, ^37^, ^38^ where HIC is measured in mg/g dry weight.

(9) $$R_{2}=\frac{1}{T_{2}}=\left(6.88+26.06{\left[\mathrm{HIC}\right]}^{0.701}-0.438{\left[\mathrm{HIC}\right]}^{1.402}\right)\times 1.47-2.2/s.$$

All images were acquired in a single transverse slice positioned between the 10th and 11th thoracic vertebrae, during a breath‐hold. Breath‐held localized spectroscopy was performed in a voxel placed in the right lobe of the liver, away from the margin of the liver and from major blood vessels and bile ducts, making sure the voxel lay in the plane used for imaging. Breath‐hold lengths varied between 9 and 15 s for imaging and were 12 s for the long‐TR spectroscopy acquisition and 21 s for the multiple‐TR, multiple‐TE acquisition.

#### 2.2.3 The MOLLI water *T*_1_ determination algorithm

Time‐varying magnetization of water and fat were simulated using Brian Hargreaves' Bloch equation simulator (http://mrsrl.stanford.edu/~brian/blochsim/). A version of the liver model introduced by Tunnicliffe et al.^16^ was used. This model comprises an intracellular and an extracellular space, with the extracellular space divided into an interstitial fluid pool and a blood pool in fast exchange. This liver model was extended with an additional fat component, and volume fractions of the extracellular fluid and the intracellular compartment were adjusted to reflect the fat fraction of the liver.

Exchange effects (both water exchange between intra‐ and extracellular spaces and magnetization transfer) were previously shown to have little influence on the simulation results,^16^ so the simulations presented here exclude exchange effects. Methods and results showing that exchange effects have little impact on these results can be found in the Supporting Information.

Fat was again characterized using six spectral peaks, with the chemical shift offsets relative to water and relative amplitudes given by Hamilton et al.^36^ *T*_1_ and *T*_2_ values of different lipid spectral peaks were fixed to values measured in the peanut oil spectra collected with the multiple‐TR, multiple‐TE STEAM sequence, except for the 2.76, 2.1, 1.3 and 0.9 ppm peaks of the human liver fat, for which *T*_2_ values reported by Hamilton et al.^36^ were used. *T*_1_ of the methylene fat peak at 1.3 ppm was quantified in patients with PDFF >5% using the multiple‐TR STEAM ^1^H MRS sequence, and a value averaged over subjects was used in the simulations. Table 2 summarizes parameters used for the simulation.

**Table 2:** Simulation parameters for the liver model at 3 T

**Parameter**	**Value**
PDFF	PDFF as measured by ^1^H STEAM MRS
*v*_c_	1 − *v*_E_ − PDFF
*v*_E_	Simulated from 0.25 to (0.95 − PDFF)
*v*_B_	$\frac{1-v_{E}-\text{PDFF}}{3}$
*v*_I_	*v*_E_ − *v*_B_
*f* [Hz]	As determined from the multiple‐echo GRE sequence
Fat chemical shifts [ppm]	As determined from the multiple‐TR, multiple‐TE STEAM ^1^H MRS sequence of peanut oil^a^
*T*_1fat_ [s]	*T*_1_ of methylene peak determined from human ^1^H spectra. Others as determined from the multiple‐TR, multiple‐TE STEAM ^1^H MRS sequence of peanut oil^a^
*T*_2fat_ [s]	Values taken from literature^39^ where available or from peanut oil spectra^a^
*R*_2E_ [1/s]	$\frac{3.64v_{B}+2.9v_{I}}{v_{E}}+26.82\;\mathrm{HI}C^{0.701}-0.451\;\mathrm{HI}C^{1.402}$
*R*_2C_ [1/s]	11.0 + 46.0 HIC^0.701^ − 0.773 HIC^1.402^
*R*_1E_ [1/s]	$\frac{0.518v_{B}+0.44v_{I}}{v_{E}}+0.029\;\mathrm{HIC}$
*R*_1C_ [1/s]	1.6 + 0.029 HIC

*v*_(C, E, B, I)_, volume fractions of the intracellular pool, extracellular fluid, blood, and interstitial fluid; *f*, off‐resonance frequency. Both fat fraction and volume fractions are unitless. *R*_2(E, C)_, transverse relaxation rates of extracellular fluid and intracellular pool; *R*_1(E, C)_, longitudinal relaxation rates of extracellular fluid and intracellular pool.

a For the full characterization of fat, see data in Table 3.

Longitudinal and transverse relaxation rates of non‐fatty compartments and their iron dependence were the same as described in the main simulation by Tunnicliffe et al.^16^ and are shown in Table 2. The longitudinal relaxation rate of the intracellular compartment in the absence of iron was determined such that the simulated signal for normal HIC (1.0 mg/g dry weight),^16^ 0% fat, on resonance and normal extracellular fluid volume fraction (ECF) (0.4) had a fitted *T*_1_ value equal to the shMOLLI *T*_1_ in healthy volunteers of 717 ms.^6^

ECF was varied between 0.25 and (0.95 − PDFF) in steps of 0.01 and was used as a proxy for liver fibrosis. To have as good an approximation of the scanner measurement as possible, the shMOLLI RF pulse sequence was simulated using the exact inversion, repetition and echo times, as well as number of phase encoding lines, extracted from the patient shMOLLI images. Once separate bSSFP signals were simulated for the intracellular pool, extracellular pool and hepatic fat, they were combined as a volume fraction‐weighted sum, as given by

(10) $$S_{\mathrm{sim}}=\left(\mathrm{ECF}\;S_{e}+\left(1-\mathrm{ECF}\;S_{i}\right)\right)\left(1-\text{PDFF}\right)+\text{PDFF}\;S_{f}.$$

In this equation *S*_sim_ represents the overall simulated shMOLLI signal, *S*_e_ is the simulated extracellular signal, *S*_i_ is the simulated intracellular signal and *S*_f_ is the simulated fat signal.

As before, the optimal simulated signal was identified via the maximization problem described by Equation 3. An ROI was placed in the posterior or lateral parts of the right lobe, in such a way as to avoid vessels and bile ducts and as close as possible to the spectroscopy voxel. The ECF corresponding to the optimal signal was used to simulate a fat‐ and iron‐free signal on resonance and *T*_1_ and *T*_2_ values of the liver compartments corresponding to normal HIC. This signal was then processed using the shMOLLI conditional fitting algorithm to obtain a water shMOLLI *T*_1_ which should be independent of iron, fat and off‐resonance frequency.

In addition, in two subjects, three separate shMOLLI *T*_1_ maps were produced for a single liver slice: an iron‐corrected, on‐resonance water shMOLLI *T*_1_ (using the full processing described in the paragraph above); iron‐corrected, on resonance mixed fat‐water shMOLLI *T*_1_; and iron‐corrected, inhomogeneous *B*_0_, water shMOLLI *T*_1_. Voxel‐by‐voxel PDFF values were determined from the *T*_2_*‐IDEAL fat‐water separation method described above followed by a magnitude‐based IDEAL algorithm.^40^ For one of the participants the shMOLLI *T*_1_ map and GRE data used for this experiment were collected after a linear frequency change was induced in the right–left direction by changing the value of the shim gradient in the *x* direction.

**Figure 1:**
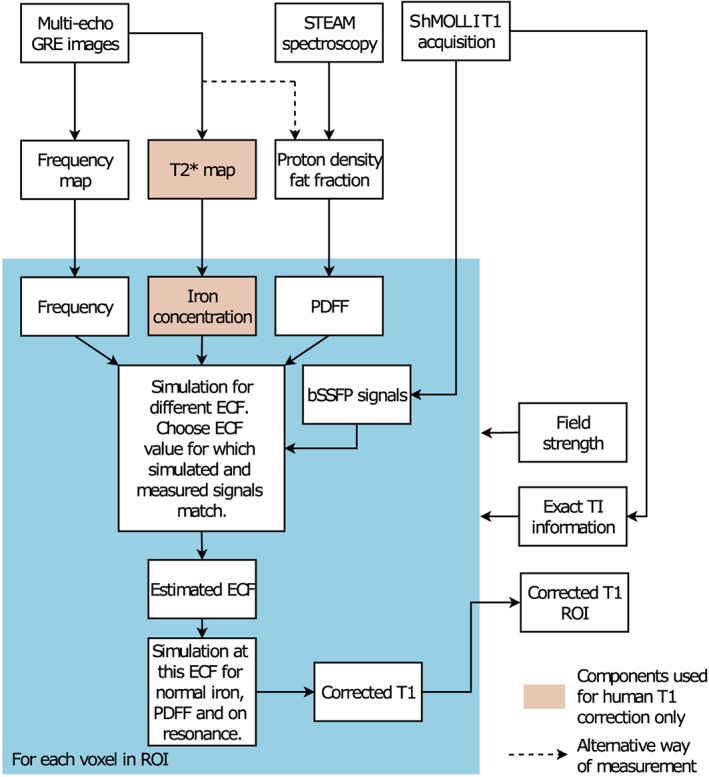
Block diagram of the water shMOLLI *T*_1_ determination algorithm. In addition to the shMOLLI *T*_1_ map, a multiple‐echo GRE image set and a STEAM ^*1*^H spectrum were also collected to derive a *B*_0_ field map of the imaged slice and the PDFF. When the algorithm was used to determine water shMOLLI *T*_1_ values in patients, the GRE images were also used to generate *T*_2_* values and thus HIC values. Knowledge of field strength was necessary for the correct modelling of lipid peaks and the effects of iron. The actual (heart‐rate‐dependent) inversion times were used as in the imaging experiments to eliminate possible errors caused by variable heart rates^2^

The mechanisms used for both phantom and patient on‐resonance water MOLLI *T*_1_ are summarized in a block diagram shown in Figure 1.

The liver model was verified against a forward simulation of patient shMOLLI *T*_1_ measurements by simulating the extracellular, intracellular and fat bSSFP signals. The extracellular volume fraction for each patient was estimated based on the spectroscopically measured water *T*_1_ and using the relationship between blood volume fraction and interstitial volume fraction as given in Table 2 and given *T*_1_ of intra‐ and extracellular compartments. *T*_1_ and *T*_2_ for the simulations were determined based on Table 2 for the intracellular and extracellular compartments, given the patient's measured HIC. The fat signal was simulated using the *T*_1_ and *T*_2_ values in Table 3. These simulated signals were combined as a volume‐fraction weighted sum and fit using the shMOLLI algorithm to produce the expected shMOLLI *T*_1_ given this measured spectroscopic *T*_1_.

**Table 3:** Fat peak parameters used in the Bloch simulation of the shMOLLI sequence. *T*_1_ and *T*_2_ values of fat peaks in peanut oil were measured at room temperature (20°C) for the phantom work and at human body temperature (37°C) for human experiments

			**Peanut oil (*T* = 20°C)**		**Participants (*T* = 37°C)**	
Peak number	Chemical shift (relative to tetramethylsilane) [ppm]	Relative amplitude [a.u.]	*T*_1_ ± SD [ms]	*T*_2_ ± SD [ms]	*T*_1_ ± SD [ms]	*T*_2_ ± SD [ms]
1	0.90	0.087	264 **±** 20	54 ± 3	333 ± 12^a^	83^b^
2	1.30	0.693	291 **±** 21	61 ± 1	312 ± 17^c^	62^b^
3	2.10	0.128	231 **±** 18	52 ± 3	254 ± 8^a^	52^b^
4	2.76	0.004	248 **±** 25	61 ± 5	273 ± 8^a^	51^b^
5	4.30	0.039	193 **±** 14	43 ± 2	228 ± 8^a^	49 ± 3^a^
6	5.30	0.049	285 **±** 15	48 ± 2	332 ± 15^a^	50 ± 4^a^

a As measured in peanut oil.

b As reported by Hamilton et al.^36^

c As measured in livers of participants.

### 2.3 Statistical analysis

Correlation between simulated and measured shMOLLI *T*_1_ values of phantoms, as well as correlation of iron‐corrected shMOLLI *T*_1_ and iron‐, fat‐ and off‐resonance‐independent water shMOLLI *T*_1_ values with spectroscopically determined water *T*_1_ values in patients, was evaluated using Pearson's correlation coefficient. Bland–Altman plots^41^ were built to evaluate agreement between water *T*_1_ values derived from STEAM spectroscopy and iron‐corrected shMOLLI *T*_1_ values, and between water *T*_1_ values derived from STEAM spectroscopy and iron‐, fat‐ and off‐resonance‐independent water shMOLLI *T*_1_ values. A left‐tailed *F*‐test was used to evaluate the change in the variance of *T*_1_ differences shown on the Bland–Altman plots. All statistical hypothesis testing was performed at significance level *α* = 0.05.

## 3 RESULTS

### 3.1 Phantom experiments

Table 3 summarizes the results of measurements made using the 100% peanut oil phantom. These values were used to characterize fat in the phantom correction algorithm.

**Figure 2:**
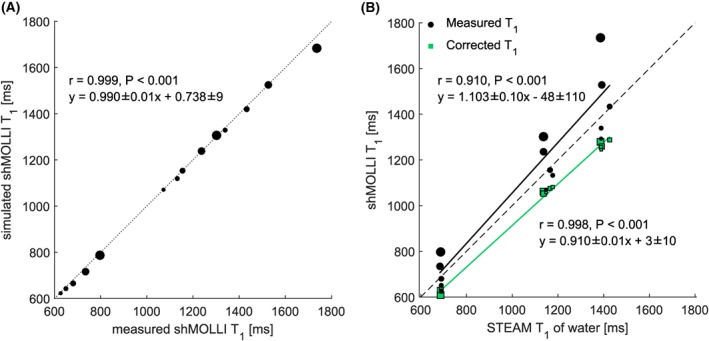
A, “Forward” shMOLLI simulation of the phantoms shows excellent agreement with measured shMOLLI *T*_1_ values. B, Correlation between shMOLLI *T*_1_ and spectroscopy‐based *T*_1_ increases after removing the effects of fat. It is expected to obtain shMOLLI *T*_1_ values lower than those obtained from spectroscopy after the determination of water shMOLLI *T*_1_ values.^32^ Points on both graphs are size‐coded as a function of phantom PDFF

**Figure 3:**
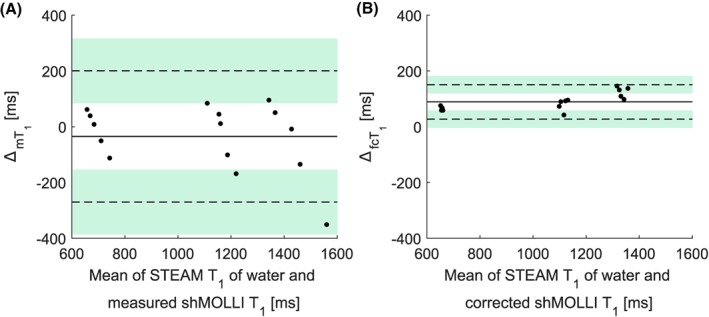
Bland–Altman plots showing phantom data before (A) and after (B) applying the water shMOLLI *T*_1_ determination algorithm. The near‐zero bias in A is due to the increased shMOLLI *T*_1_ values observed at high fat fractions, while a bias of 89 ms in B can be explained by the expected^2^ underestimation of *T*_1_ values by the shMOLLI method. An *F*‐test performed on the two differences shown in A and B revealed that the variance of the measurements was statistically significantly decreased after removing the effects of fat, iron and off‐resonance frequency (*F* = 15.003, *P* < 0.001). $\Delta_{mT_{1}}$ is the difference between STEAM *T*_1_ and the measured shMOLLI *T*_1_; $\Delta_{\mathrm{fc}T_{1}}$ is the difference between STEAM *T*_1_ and the fat‐independent water shMOLLI *T*_1_

Table 4 shows PDFF, *T*_1_, *T*_2_ and shMOLLI *T*_1_ of individual fat‐water phantoms. Simulated and measured phantom shMOLLI *T*_1_ values showed good agreement (*r* = 0.999, *P* < 0.001), as reflected by Figure 2A. Figure 2B shows the corrected on‐resonance water shMOLLI *T*_1_ in comparison with the measured shMOLLI *T*_1_ values. Fat‐dependent shMOLLI *T*_1_ had a clear systematic dependence on PDFF and a weaker correlation (*r* = 0.910, *P* < 0.001) with spectroscopy‐based *T*_1_ than fat‐independent water shMOLLI *T*_1_ (*r* = 0.998, *P* < 0.001). On‐resonance water shMOLLI *T*_1_ had significantly (*F* = 15.003, *P* = 0.0046) lower variance than measured shMOLLI *T*_1_ of the difference between phantom water *T*_1_ values measured by STEAM and by shMOLLI, as shown in Figure 3.

**Table 4:** Baseline parameter values for fat‐water phantoms, as measured with multiple‐contrast spin echo, shMOLLI and multiple‐TR STEAM ^*1*^H MRS. Water *T*_2_ and shMOLLI *T*_1_ were averaged over circular regions of interest on corresponding maps

**NiCl**_**2**_ **concentration [mM]**	**PDFF [%]**	**STEAM *T***_**1**_ **of water ± SD [ms]**	***T***_**2**_ **of water ± SD [ms]**	**shMOLLI *T***_**1**_ **± SD [ms]**
0.45	0.0	1390 ± 30	78 ± 3	1294 ± 31
	7.0	1386 ± 24	a	1340 ± 29
	10.3	1425 ± 35	a	1433 ± 38
	15.6	1392 ± 29	a	1527 ± 42
	26.8	1386 ± 17	a	1736 ± 62
0.73	0.0	1148 ± 29	82 ± 2	1071 ± 18
	7.3	1177 ± 34	a	1132 ± 25
	11.1	1169 ± 20	a	1156 ± 21
	16.0	1136 ± 30	a	1236 ± 31
	24.7	1135 ± 28	a	1303 ± 39
1.61	0.0	690 ± 23	89 ± 5	627 ± 7
	6.8	690 ± 29	a	651 ± 7
	12.6	689 ± 27	a	680 ± 8
	21.5	684 ± 20	a	735 ± 11
	27.0	687 ± 16	a	798 ± 12

a As the MCSE experiment could not be used to determine water *T*_2_ when mixed with fat, we have used the same water *T*_2_ within a NiCl_2_ concentration group as the *T*_2_ measured in the 0% fat phantom of each group.

### 3.2 Patient studies

**Figure 4:**
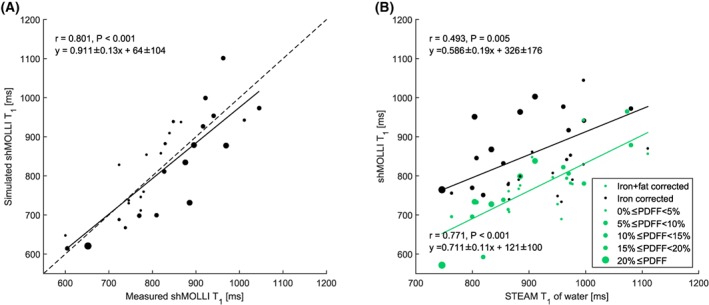
A, “Forward” simulation of patient shMOLLI *T*_1_ measurements. B, Correlation between water shMOLLI *T*_1_ values and spectroscopically measured *T*_1_ of the water in the liver increased after extending the initial iron‐only correction to remove the effects of iron, fat and off‐resonance

**Figure 5:**
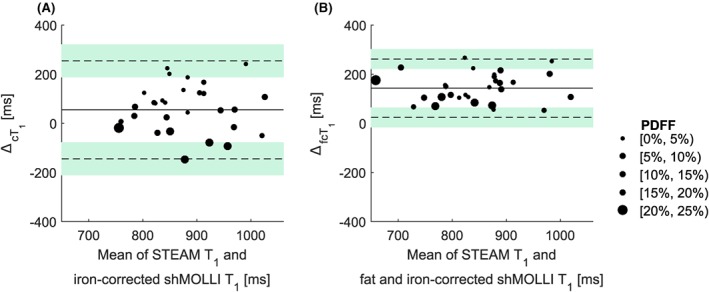
Bland–Altman plots of the iron‐corrected (A) and iron‐, fat‐ and off‐resonance‐independent (B) patient data reveal a statistically significant (*F* = 0.3448, *p* = 0.0023) reduction of the variance in the difference between STEAM *T*_1_ and shMOLLI *T*_1_ values after removing the effects of iron, fat and *B*_0_ inhomogeneities and remove the systematic trend for higher fat to give higher shMOLLI *T*_1_. As in the case of phantoms, the large bias in B is caused by the previously shown^2^ underestimation of *T*_1_ values by the shMOLLI method. $\Delta_{cT_{1}}$ is the difference between the STEAM *T*_1_ of the water in the liver and the iron‐corrected shMOLLI *T*_1_; $\Delta_{\mathrm{fc}T_{1}}$ is the difference between the STEAM *T*_1_ of the water in the liver and the iron‐, fat‐ and off‐resonance‐independent water shMOLLI *T*_1_

Of 30 patients, 19 (63.3%) had hepatic steatosis (PDFF >5%). The patients' fat fractions ranged from 1.53% to 20.33% and *T*_2_* values ranged from 6.3 ms to 37.9 ms (HIC range 0.6768 mg/g dry weight‐2.357 mg/g dry weight). Figure 4A shows the results of the forward simulation, while Figure 4B shows the results of the fat correction in patients. While iron‐corrected shMOLLI *T*_1_ values correlated moderately (*r* = 0.493, *P* = 0.005) with spectroscopically measured water *T*_1_ values, the fat‐independent water shMOLLI *T*_1_ determination algorithm yielded a stronger correlation with STEAM *T*_1_ values (*r* = 0.771, *P* < 0.001). Similarly, the large variability between STEAM and iron‐corrected shMOLLI *T*_1_ introduced by the effect of fat is significantly reduced by the water shMOLLI *T*_1_ determination algorithm (*F* = 0.3448, *P* = 0.0023), as seen in Figure 5B.

Relaxation times of individual fat peaks used for modelling the fat compartment are summarized in Table 3.

Table 5 contains linear regression coefficients for the dependence of shMOLLI *T*_1_ on water STEAM *T*_1_, PDFF and *R*_2_* (=1/*T*_2_*) values as well as *P*‐values associated with these coefficients, showing that on‐resonance water shMOLLI *T*_1_ values do not show statistically significant dependence on fat.

**Table 5:** Linear regression correlation coefficients for shMOLLI *T*_1_ dependence on water STEAM *T*_1_, PDFF and liver *R*_2_*, determined from original measured shMOLLI *T*_1_ values, iron‐corrected shMOLLI *T*_1_ values, and iron‐, fat‐, and off‐resonance‐independent water shMOLLI *T*_1_ values. Coefficients are shown as estimate ± standard error

	**STEAM *T***_**1**_ **coefficient [ms shMOLLI *T***_**1**_ **/ms STEAM *T***_**1**_ **]**	***P*‐value**	**PDFF coefficient [ms shMOLLI *T***_**1**_ **/% PDFF]**	***P*‐value**	***R***_**2**_ * coefficient [ms shMOLLI *T*_**1**_ **/(1/s) *R***_**2**_ *]	***P*‐value**
Measured shMOLLI *T*_1_	0.5 ± 0.2	0.0051	11.7 ± 2.5	< 0.0010	−1.7 ± 0.4	<0.0010
Iron‐corrected shMOLLI *T*_1_	0.8 ± 0.1	<0.0010	13.9 ± 2.1	< 0.0010	−0.4 ± 0.4	0.3544
Iron‐, fat‐ and off‐resonance‐independent water shMOLLI *T*_1_	0.7 ± 0.1	<0.0010	2.9 ± 1.7	0.1040	−0.6 ± 0.3	0.0400

**Figure 6:**
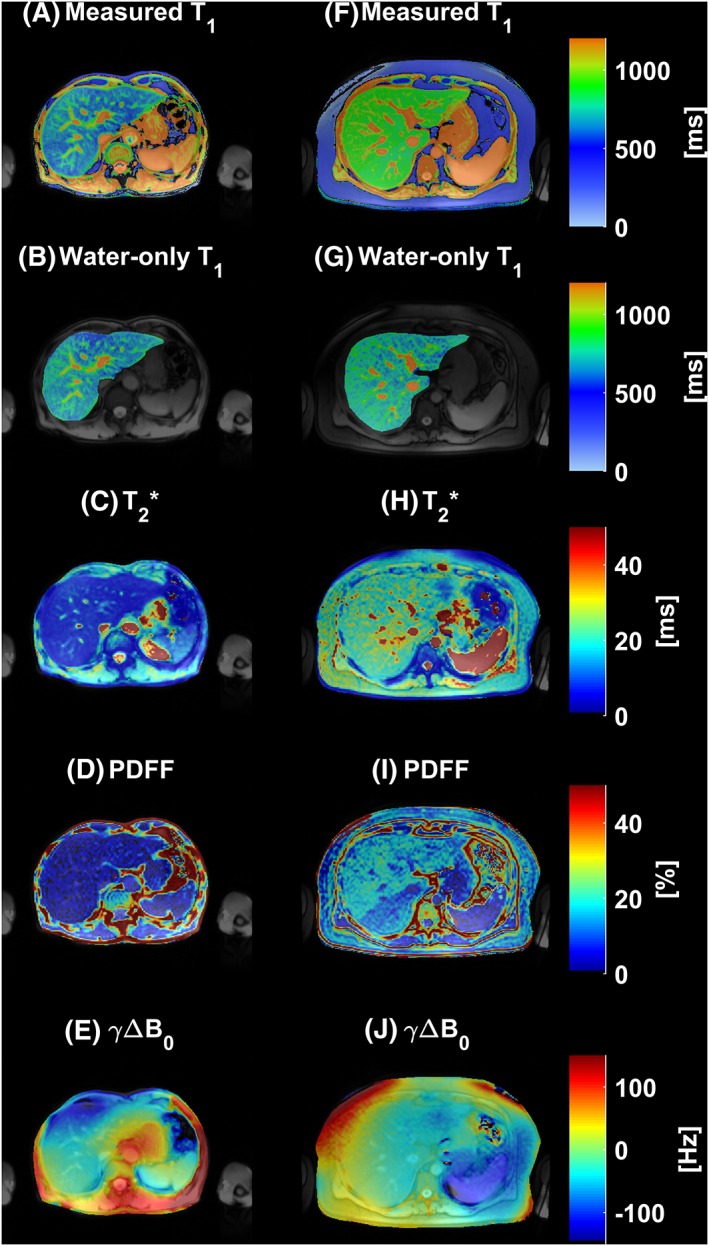
A, F, Two examples of liver shMOLLI *T*_1_ maps affected by iron, fat and *B*_0_ inhomogeneity. B, G, Normal liver *T*_1_ maps resulted after removing the effects of fat, iron and *B*_0_ inhomogeneity. C, H, The calculated *T*_2_* maps show a homogeneous distribution of increased (mean HIC = 2.69 mg/g dry weight) (C) and normal iron concentrations (mean HIC = 1.16 mg/g dry weight) (H). D, E, I, J, Full PDFF (D, mean PDFF = 8.35%; I, mean PDFF = 15.83%) and *B*_0_ field maps (E, mean *γ ΔB*_0_ = 1.78 Hz; J, mean *γ ΔB*_0_ = −8.81 Hz) are also shown

Figure 6 shows the results of applying the algorithm pixel by pixel over a single‐slice liver shMOLLI *T*_1_ map of two patients. The liver in the right‐hand column had, on average, HIC = 1.16 mg/g dry weight, PDFF = 15.83% and a linearly varying frequency offset in the left–right direction with frequency offsets ranging from −50 Hz to 35 Hz, which was artificially applied by manually modifying the *B*_0_ shim. The liver in the left column had an average HIC = 2.69 mg/g dry weight and PDFF = 8.35%.

**Figure 7:**
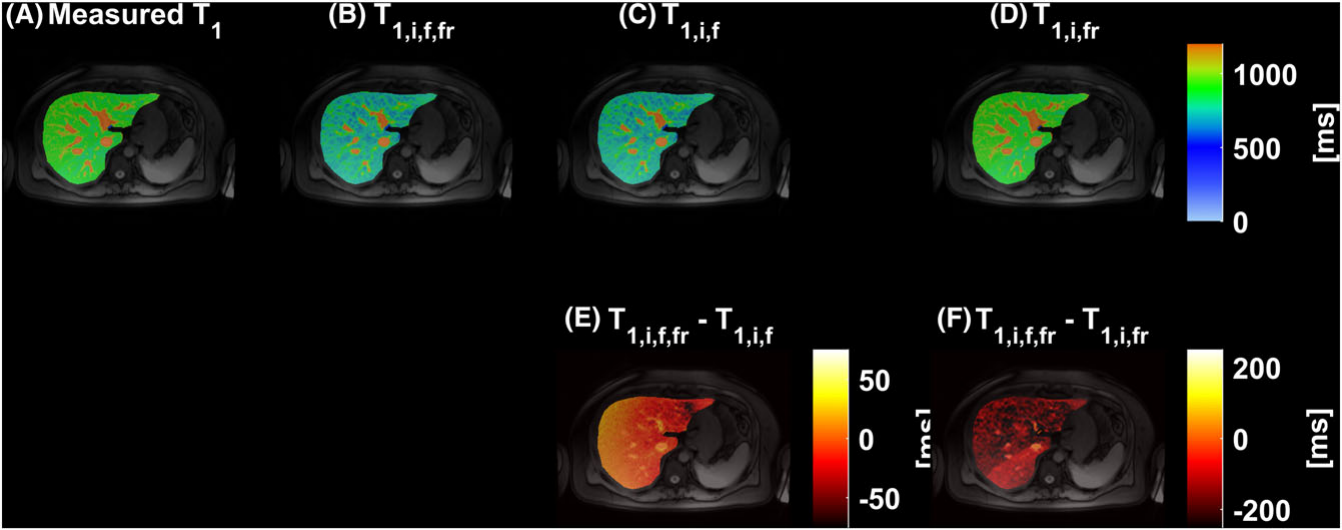
Three models were used in total to determine water‐only shMOLLI *T*_1_ based on a measured shMOLLI *T*_1_ map (A). In addition to the full model of iron, fat and off‐resonance effects (B), two additional models were implemented: one without accounting for *B*_0_ inhomogeneity and another without accounting for fat. C, The resulting *T*_1_ map after the effects of iron and fat have been removed; E, the difference between the *T*_1_ map obtained by using the full model and this alternative corrected *T*_1_ map. D, Removing the effects of iron and *B*_0_ inhomogeneity only yields higher‐than‐normal *T*_1_ values. The difference between this alternative *T*_1_ map and the water shMOLLI *T*_1_ map determined using the full model (F) is explained by the effects of the fat

The different effects on the overall on‐resonance water‐only *T*_1_ determination are shown in Figure 7 for the participant who had the *x* shim manually changed over the liver. Figures 7B, 7C and 7D show the results of the algorithm with three different models applied on the same *T*_1_ map of the liver: full modelling of the effects of iron, fat and *B*_0_ inhomogeneity (Figure 7B), a model of iron and fat effects (Figure 7C) and a model containing effects from iron and *B*_0_ inhomogeneity (Figure 7D). The maps in the second row of Figure 7 show the *T*_1_ difference between the complete model and the partial models. As expected, a linear variation in off‐resonance frequencies over a range including both positive and negative values caused both an increase and a decrease of *T*_1_ values,^10^ in this case by up to 50 ms. This under‐ and overestimation of *T*_1_ values is reflected in the difference between the iron‐, fat‐ and off‐resonance‐independent water shMOLLI *T*_1_ map and the iron‐ and fat‐independent *T*_1_ map of Figure 7E. When removing the effects of iron and off‐resonance frequencies only, the effects of fat lead to higher‐than‐expected shMOLLI *T*_1_ values, as shown in Figure 7F, in this case by up to 250 ms.

## 4 DISCUSSION

This study describes an extension of the liver model proposed by Tunnicliffe et al.^16^ to include a variable sized fat compartment and a frequency offset calculated from the GRE data used for *T*_2_* mapping, thus obtaining a model reflecting the effects of iron, fat and off‐resonance frequencies on *T*_1_ measured by MOLLI variants. This model then allowed the identification of an on‐resonance water shMOLLI *T*_1_ value: the value that would have been measured if subjects had 0% PDFF, and normal HIC (i.e. 1 mg/g dry weight) and were imaged in a perfectly homogeneous static magnetic field. The results of the on‐resonance water shMOLLI *T*_1_ determination algorithm showed excellent correlation with spectroscopically determined water *T*_1_ values in phantoms, and increased statistically significant correlation in human participants, when compared with iron‐corrected shMOLLI *T*_1_, and reduced variance of the difference between STEAM *T*_1_ of water and water shMOLLI *T*_1_. While we report our results for the particular case of using the shMOLLI *T*_1_ mapping method, the water *T*_1_ determination algorithm is equally applicable to other variants of the MOLLI acquisition method, as long as the specific timing is included in the Bloch simulations..

In addition to the assumptions of Tunnicliffe et al. regarding the homogeneous distribution of iron between the liver parenchyma and extracellular fluid, we further assumed that fat droplets in hepatocytes appear in a macrovesicular form, which is mostly prevalent in NAFLD,^42^ thus not affecting the tumbling rate of water molecules in hepatocytes. We have not explored possible different relaxation mechanisms in the presence of microvesicular fat.

A further assumption was that the *T*_1_ and *T*_2_ relaxation times of lipid protons are independent of iron. It has been shown previously that the *T*_2_* of both water and fat is affected by iron.^43^ However, fat diffuses much more slowly than water molecules because of the high molecular weights of constituent triglycerides; therefore, their transverse *T*_2_ relaxation will be affected less by local field inhomogeneities. The independence of longitudinal relaxation of fat on iron is guaranteed by the lack of magnetization transfer between lipid macromolecules and water.^44^

In the *T*_2_*‐IDEAL model we assumed equal *T*_2_* values for fat and water to ensure numerical stability of the fitting. However, the *T*_2_* values of these two species might not be identical due to a susceptibility difference between them.^45^ Errors in fat quantification due to this simplification appear in Bydder et al^45^ to be small enough that they would not have caused substantial changes in our proposed water‐*T*_1_ determination algorithm, particularly when PDFF is lower than 21% as shown here.

Our proposed algorithm can be implemented in at least two ways. Running the algorithm “on‐line”, as we have done here, by simulating patient signals every time an on‐resonance water shMOLLI *T*_1_ is needed, is one option. Alternatively, one can build a look‐up table a priori and look up the measured *T*_1_ values along axes of off‐resonance frequency, PDFF and HIC and then project the *T*_1_ value along the identified ECF axis to 0 Hz off‐resonance frequency, 1 mg/g dry weight HIC and 0% PDFF. In this study, we have used ^1^H STEAM MRS to determine PDFF locally and determine the water shMOLLI *T*_1_ over a spectroscopic voxel of interest, as well as a complex‐based *T*_2_*‐IDEAL fat‐water separation method with field map estimation followed by a magnitude‐based IDEAL fat‐water separation method to map fat fractions over the imaging slice, allowing determination of water shMOLLI *T*_1_ values over the whole liver slice.

Human hepatic lipid was modelled using a hybrid model comprising chemical shift, relative amplitude, *T*_1_ and *T*_2_ values taken from human hepatic lipid literature and—due to its similarity to subcutaneous fat^23^—peanut oil spectroscopy measurements. However, it has been shown that the hepatic fat spectrum is different from the spectrum of abdominal subcutaneous adipose tissue^46^, ^47^; therefore, errors in the correction due to the inaccurate spectral modelling might reveal themselves at higher fat fractions where small fat peaks have a larger contribution to the overall signal. Furthermore, *T*_1_ and *T*_2_ relaxation times of individual fat peaks of the peanut oil spectra were determined using mono‐exponential fitting, although it is known that *J*‐coupling effects disturb the pure mono‐exponential behaviour of these peaks. Since *J*‐coupling effects were not included in any of our Bloch equation simulations and approximate relaxation times of fat peaks gave satisfactory results in phantoms, we chose not to include *J*‐coupling effects in the patient study.

Validation of the whole multi‐compartment model as an extension of the model described by Tunnicliffe et al.^16^ is difficult, due to the challenge of building phantoms with multiple sub‐voxel water compartments, mimicking liver tissue. However, we built an array of fat‐water phantoms with two compartments and proved the validity of fat modelling and the water shMOLLI *T*_1_ determination algorithm using these phantoms.

While the *T*_1_ value of the tissue water can be determined spectroscopically,^29^ there have been attempts to also produce *T*_1_ maps of tissue that are independent of fat. Hoad et al.^48^ reported results of a liver imaging study involving participants with fibrotic livers, in which a liver *T*_1_ determined using an IR SE‐EPI (inversion recovery spin echo echo‐planar imaging) sequence was shown to be independent of fat and to correlate well with fibrosis. Pagano et al.^49^ proposed an IDEAL‐*T*_1_ technique that is a modified version of SASHA,^50^ collecting images at several echo times for each saturation time over two breath‐holds. A different approach was described by Nezafat et al.^51^ that uses a modified version of the slice‐interleaved *T*_1_ (STONE) sequence^52^ with spectrally water‐selective inversion pulses instead of adiabatic inversion pulses. A more recent method uses the water‐only Look‐Locker inversion recovery (WOLLI) sequence,^53^ which produces water‐only *T*_1_ maps by using hypergeometric inversion pulses to selectively invert water. However, our proposed method of determining water (sh)MOLLI *T*_1_ maps independent of the effects of fat also shows promise, as (sh)MOLLI is widely available and water shMOLLI *T*_1_ values can be extracted retrospectively.

One of the limitations of this study is that water shMOLLI *T*_1_ values of only 30 patients were determined, and only 19 patients had clinically significant fat fractions in their livers, i.e. PDFF > 5%. The range of fat fractions in our patient population was 1.53%–20.33%; therefore, the correction method was not validated in patients with higher liver fat fractions, although it is known that the concentration of fat in the liver can reach about 45%.^54^, ^55^ We also considered only single‐slice coverage of the liver, along with performing ROI‐based analyses instead of mapping over the acquired slice in all but two cases. The multivariable regression analysis of on resonance water *T*_1_ values of patients shows an unexpected, though marginal, statistically significant dependence on iron, but this was due to a single participant with high iron concentration and a very low on‐resonance water *T*_1_. We also note that the forward simulation of patient shMOLLI *T*_1_ did not completely recover the measured shMOLLI *T*_1_. However, the gradient and intercept in Figure 4a are statistically equivalent to 1 and 0, and simulation results were spread relatively uniformly around the unity line with respect to fat and iron concentration distributions. We believe that this suggests that the remaining variability in water shMOLLI *T*_1_ values determined by our algorithm are due to variability of the population and random measurement errors in the input model parameters, rather than any systematic errors in the approach, in which case the simulation results would be either predominantly above or below the line of unity. One principal source of random error is the recently reported dependence of multiple‐TR, multiple‐TE STEAM *T*_1_ values on *B*_1_ inhomogeneities.^56^ We note that any correction process will necessarily add some noise to the data. However, the mean perpendicular distance of forward‐simulated shMOLLI *T*_1_ values from the regression line (*d* = 41 ms) is comparable to the standard deviation observed in healthy volunteers lacking pathology (*σ* = 49 ms), implying that any additional variability added by the correction process is not substantially greater than the usual variation within a population.

In conclusion, we have described a four‐compartment model of the liver capable of characterizing the combined effect of fat, iron and off‐resonance frequency on shMOLLI *T*_1_ values. This enables the extraction of an on‐resonance water‐only shMOLLI *T*_1_, which may help to better characterize fibro‐inflammatory liver disease using *T*_1_ mapping.

## FUNDING INFORMATION

The research was funded by a UK Medical Research Council Doctoral Training Award (MR/K501256/1), a Scatcherd European Scholarship, the RDM Scholars Programme and the National Institute for Health Research (NIHR) Oxford Biomedical Research Centre Programme. The views expressed are those of the authors and not necessarily those of the NHS, the NIHR or the Department of Health. This work is the subject of a priority UK patent application.

## Supporting information


**Figure S1** Correcting for fat increased the correlation between STEAM T_1_ of the water in the liver and shMOLLI T_1_ values. PDFF of individual data points is encoded in point size.
**Figure S2** Bland–Altman plots showing patient data after iron‐only (a) and after (b) iron‐, fat‐ and off‐resonance correction. The underestimation of T_1_ values by the shMOLLI method explains the bias on the agreement plots. An F‐test performed on the two differences shown in the subfigures revealed that the variance of measurements significantly decreased after the correction. $\Delta_{cT_{1}}$ is the difference between STEAM T_1_ of the water in the liver and the iron‐corrected shMOLLI T_1_; $\Delta_{\mathrm{fc}T_{1}}$ is the difference between STEAM T_1_ of the water in the liver and the iron‐, fat‐ and off‐resonance‐corrected shMOLLI T_1_
**Figure S3** Bland–Altman plot showing that the MT‐enabled correction is generally underestimating the non‐MT‐enabled correction. *fcT*_1_ is a non‐MT‐enabled iron‐corrected water shMOLLI T_1_ with fat and B_0_ inhomogeneity modelling and $\mathrm{fc}T_{1_{\mathrm{MT}}}$ is an MT‐enabled iron‐corrected water shMOLLI T_1_ with fat and B_0_ inhomogeneity modelling
**Table SI**1 Simulation parameters for the liver model at 3 T. PDFF: proton density fat fraction, v_(S, L, E, B, I)_: volume fractions of the intracellular semisolid pool, intracellular liquid pool, extracellular fluid, blood, and interstitial fluid, f: off‐resonance frequency. Both fat fraction and volume fractions are unitless. R_2(B, I, E, S, L)_: transverse relaxation rates of blood, interstitial fluid, extracellular fluid, semisolid intracellular pool and liquid intracellular pool, R_1(B, I, E, S, L)_: longitudinal relaxation rates of blood, interstitial fluid, extracellular fluid, semisolid intracellular pool and liquid intracellular pool.
**Table S2** Linear regression correlation coefficients for water STEAM T_1_, PDFF and liver R_2_*, determined from original measured shMOLLI T_1_ values, iron‐corrected shMOLLI T_1_s with MT and iron‐corrected water shMOLLI T_1_ values with MT and fat and B_0_ inhomogeneity modelling. Coefficients are shown as estimate ± standard error.
