# Supplementary material for: Mapping tissue water T 1 in the liver using the MOLLI T 1 method in the presence of fat, iron and B 0 inhomogeneity
Source: NMR Biomed. 2018 Nov 21;32(2):e4030. doi: 10.1002/nbm.4030 (PMC6492199; doi:10.1002/nbm.4030)
Supplement: Supplementary file 1 — Figure S1 Correcting for fat increased the correlation between STEAM T1 of the water in the liver and shMOLLI T1 values. PDFF of individual data points is encoded in point size. Figure S2 Bland–Altman plots showing patient data after iron‐only (a) and after (b) iron‐, fat‐ and off‐resonance correction. The underestimation of T1 values by the shMOLLI method explains the bias on the agreement plots. An F‐test performed on the two differences shown in the subfigures revealed that the variance of measurements significantly decreased after the correction. ΔcT1 is the difference between STEAM T1 of the water in the liver and the iron‐corrected shMOLLI T1; ΔfcT1 is the difference between STEAM T1 of the water in the liver and the iron‐, fat‐ and off‐resonance‐corrected shMOLLI T1 Figure S3 Bland–Altman plot showing that the MT‐enabled correction is generally underestimating the non‐MT‐enabled correction. fcT1 is a non‐MT‐enabled iron‐corrected water shMOLLI T1 with fat and B0 inhomogeneity modelling and fcT1MT is an MT‐enabled iron‐corrected water shMOLLI T1 with fat and B0 inhomogeneity modelling Table SI1 Simulation parameters for the liver model at 3 T. PDFF: proton density fat fraction, v(S, L, E, B, I): volume fractions of the intracellular semisolid pool, intracellular liquid pool, extracellular fluid, blood, and interstitial fluid, f: off‐resonance frequency. Both fat fraction and volume fractions are unitless. R2(B, I, E, S, L): transverse relaxation rates of blood, interstitial fluid, extracellular fluid, semisolid intracellular pool and liquid intracellular pool, R1(B, I, E, S, L): longitudinal relaxation rates of blood, interstitial fluid, extracellular fluid, semisolid intracellular pool and liquid intracellular pool. Table S2 Linear regression correlation coefficients for water STEAM T1, PDFF and liver R2*, determined from original measured shMOLLI T1 values, iron‐corrected shMOLLI T1s with MT and iron‐corrected water shMOLLI T1 values with MT and fat and B0 i [file NBM-32-na-s001.docx]

Supplementary Material

Mapping tissue water T_1_ in the liver using the MOLLI T1 method in the presence of fat, iron and B_0_ inhomogeneity

# **Methods**

Table SI1 summarizes the simulation parameters used for a water shMOLLI T_1_ determination method that includes exchange effects between liver compartments.

Table SI1 Simulation parameters for the liver model at 3 T. PDFF: proton density fat fraction, v_(S, L, E, B, I)_: volume fractions of the intracellular semisolid pool, intracellular liquid pool, extracellular fluid, blood, and interstitial fluid, f: off-resonance frequency. Both fat fraction and volume fractions are unitless. R_2(B, I, E, S, L)_: transverse relaxation rates of blood, interstitial fluid, extracellular fluid, semisolid intracellular pool and liquid intracellular pool, R_1(B, I, E, S, L)_: longitudinal relaxation rates of blood, interstitial fluid, extracellular fluid, semisolid intracellular pool and liquid intracellular pool.

| Parameter | Value |
| --- | --- |
| PDFF | Proton density fat fraction as measured by ^1^H STEAM MRS |
| v_S_ | $0.07\left( 1 - v_{E}- PDFF \right)$ |
| v_L_ | $0.93\left( 1 - v_{E}- PDFF \right)$ |
| v_E_ | Simulated from 0.25 to $\left( 0.95-PDFF \right)$ |
| v_B_ | $\frac{1-v_{E}-PDFF}{3}$ |
| v_I_ | $v_{E}-v_{B}$ |
| f [Hz] | As determined from the multiple-echo GRE sequence |
| Fat chemical shifts [ppm] | As determined by the multiple-TR, multiple-TE STEAM ^1^H MRS sequence of peanut oil |
| T_1fat_ [s] | T_1_ of methylene peak determined from human ^1^H spectra. Others as determined by the multiple-TR, multiple-TE STEAM ^1^H MRS sequence of peanut oil. |
| T_2fat_ [s] | Values have been taken from the literature (1). Values for some of the peaks not available in the literature were determined from peanut oil spectra. |
| R_2B_ [$s^{-1}$] | $3.64+\left( 0.7\times26.06HIC^{0.701}-0.7\times0.438HIC^{1.402} \right)\times1.47$ |
| R_2I_ [$s^{-1}$] | $2.9+\left( 0.7\times26.06HIC^{0.701}-0.7\times0.438HIC^{1.402} \right)\times1.47$ |
| R_2E_ [$s^{-1}$] | $\frac{3.64v_{B}+2.9v_{I}}{v_{E}}+26.82HIC^{0.701}-0.451HIC^{1.402}$ |
| R_2S_ [$s^{-1}$] | $\frac{1}{7.7\times{10}^{-6}}$ |
| R_2L_ [$s^{-1}$] | $11.0+46.0HIC^{0.701}-0.773HIC^{1.402}$ |
| R_1B_ [$s^{-1}$] | $0.518+0.029HIC$ |
| R_1I_ [$s^{-1}$] | $0.44+0.029HIC$ |
| R_1E_ [$s^{-1}$] | $\frac{0.518v_{B}+0.44v_{I}}{v_{E}}+0.029HIC$ |
| R_1S_ [$s^{-1}$] | 1.00 |
| R_1L_ [$s^{-1}$] | $1.6+0.029HIC$ |
| k_LS_ [s^-1^] | $3.52/v_{L}$ |
| k_SL_ [s^-1^] | $3.52/v_{S}$ |
| k_LE_ [s^-1^] | $0.52/v_{L}$ |
| k_EL_ [s^-1^] | $0.52/v_{E}$ |

The water shMOLLI T_1_ determination algorithm was built in a similar fashion to that presented in the main Methods section for patients, with the difference that instead of Bloch equations, Bloch-McConnell equations were simulated to model exchange between the semisolid and liquid pools of the intracellular compartment of the liver and the liquid pool of the intracellular compartment and the extracellular compartment.

The shMOLLI RF pulse sequence was simulated for each extracellular fluid volume fraction. Once separate signals for the semisolid intracellular pool, the liquid intracellular pool the extracellular pool and hepatic fat were simulated, they were combined to form the final bSSFP signal that was fitted using the shMOLLI conditional fitting algorithm (2).

**RESULTS**

Figure S1 presents the water shMOLLI T_1_s determined when considering the iron- and fat concentrations and B_0_ inhomogeneity alongside the results from iron-correction only. Figure S2 shows a significant (P < 0.001) drop in the variance of shMOLLI T_1_ values when modelling the fat and B_0_ inhomogeneities in addition to the iron on a Bland-Altman plot.


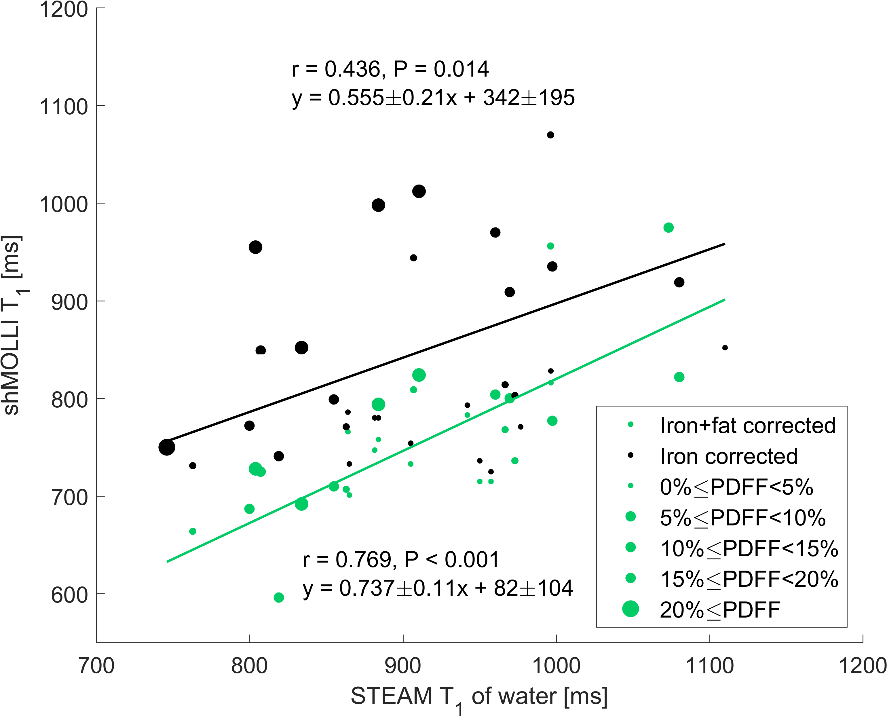


Figure S1 Correcting for fat increased the correlation between STEAM T_1_ of the water in the liver and shMOLLI T_1_ values. PDFF of individual data points is encoded in point size.


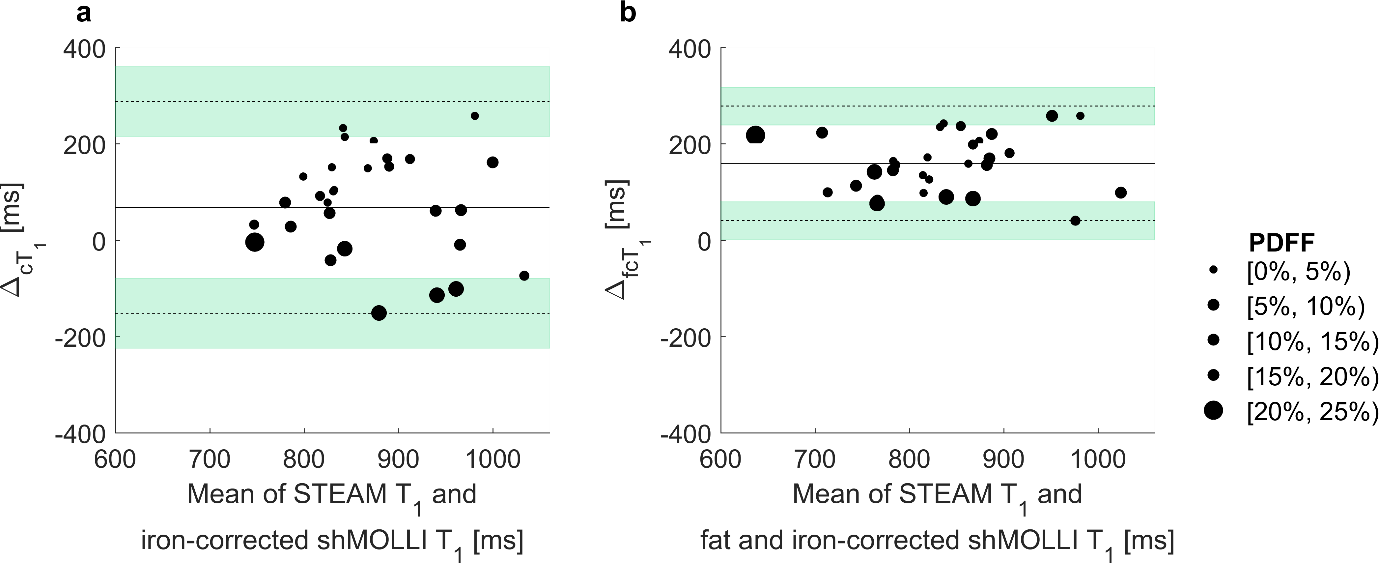


Figure S2 Bland-Altman plots showing patient data after iron-only (a) and after (b) iron-, fat- and off-resonance correction. The underestimation of T_1_ values by the shMOLLI method explains the bias on the agreement plots. An F-test performed on the two differences shown in the subfigures revealed that the variance of measurements significantly decreased after the correction. $\Delta_{cT_{1}}$ is the difference between STEAM T_1_ of the water in the liver and the iron-corrected shMOLLI T_1_; $\Delta_{fcT_{1}}$ is the difference between STEAM T_1_ of the water in the liver and the iron-, fat- and off-resonance-corrected shMOLLI T_1_

Table S2 Linear regression correlation coefficients for water STEAM T_1_, PDFF and liver R_2_*, determined from original measured shMOLLI T_1_ values, iron-corrected shMOLLI T_1_s with MT and iron-corrected water shMOLLI T_1_ values with MT and fat and B_0_ inhomogeneity modelling. Coefficients are shown as estimate ± standard error.

|  | STEAM T_1_ coefficient  [ms shMOLLI T_1_/ms STEAM T_1_] | P-value | PDFF  coefficient  [ms shMOLLI T_1_/% PDFF] | P-value | R_2_^*^  coefficient  [ms shMOLLI T_1_/s^-1^ R_2_^*^] | P-value |
| --- | --- | --- | --- | --- | --- | --- |
| Measured shMOLLI T_1_ | 0.9 ± 0.1 | <0.0001 | 13.2 ± 2.6 | <0.0001 | -1.6 ± 0.3 | <0.0001 |
| Iron-corrected shMOLLI T_1_ with MT | 0.9 ± 0.1 | <0.0001 | 14.8 ± 2.3 | <0.0001 | -0.04 ± 0.4 | 0.9249 |
| Iron-corrected water shMOLLI T_1_ with MT and fat and B_0_ inhomogeneity modelling | 0.8 ± 0.1 | <0.0001 | 2.5 ± 1.9 | 0.2028 | -0.3 ± 0.3 | 0.4323 |


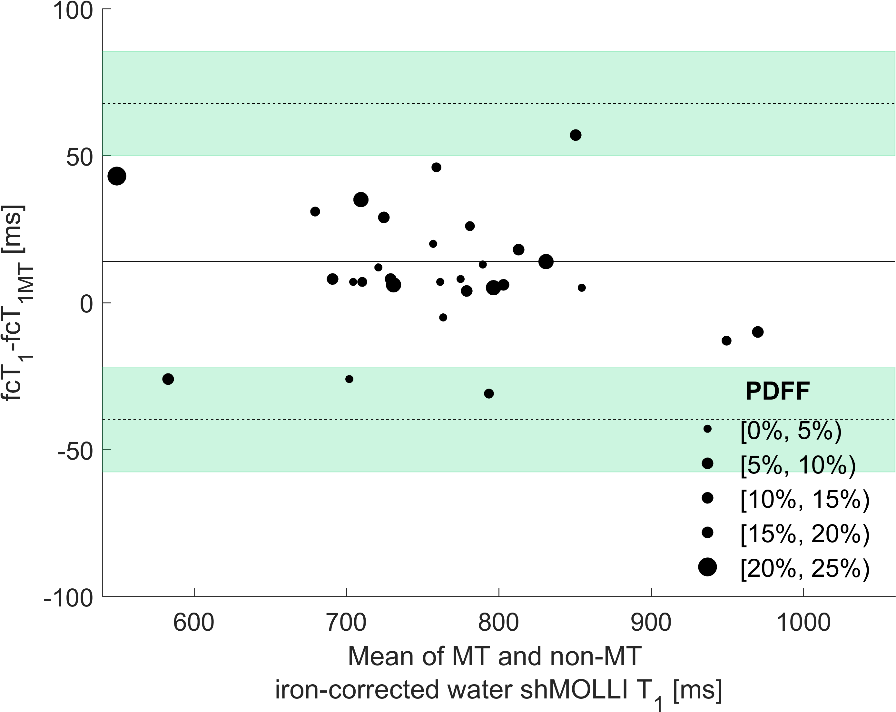


Figure S3 Bland-Altman plot showing that the MT-enabled correction is generally underestimating the non-MT-enabled correction. $fcT_{1}$ is a non-MT-enabled iron-corrected water shMOLLI T_1_ with fat and B_0_ inhomogeneity modelling and $fcT_{1_{MT}}$ is an MT-enabled iron-corrected water shMOLLI T_1_ with fat and B_0_ inhomogeneity modelling

A paired t-test between water shMOLLI T_1_s with and without MT simulation has not revealed a significant difference between the two resulting sets of water shMOLLI T_1_ values (P = 0.528). The values determined with MT simulation were numerically, but not significantly (left-tailed t-test P = 0.4589) lower than their non-MT-simulated counterparts, which has previously been shown to be the case (3) already.

Table S2 shows that all significant dependence on iron and fat is removed by our algorithm. The near-identical Pearson’s correlation coefficients of the MT- and non-MT-enabled algorithms and the Bland-Altman plot shown in figure S3 suggest that for all practical purposes the two algorithms produce very similar results.

**Supporting References**

1. Hamilton G, Schlein AN, Middleton MS, Hooker CA, Wolfson T, Gamst AC, et al. In vivo triglyceride composition of abdominal adipose tissue measured by ^1^ H MRS at 3T. J Magn Reson Imaging [Internet]. 2016 Aug [cited 2016 Sep 1]; Available from: http://doi.wiley.com/10.1002/jmri.25453

2. Piechnik SK, Ferreira VM, Dall’Armellina E, Cochlin LE, Greiser A, Neubauer S, et al. Shortened Modified Look-Locker Inversion recovery (ShMOLLI) for clinical myocardial T1-mapping at 1.5 and 3 T within a 9 heartbeat breathhold. J Cardiovasc Magn Reson [Internet]. 2010 Jan [cited 2015 Jan 12];12(1):69. Available from: http://www.jcmr-online.com/content/12/1/69

3. Tunnicliffe EM, Banerjee R, Pavlides M, Neubauer S, Robson MD. A model for hepatic fibrosis: the competing effects of cell loss and iron on shortened modified Look-Locker inversion recovery *T* _1_ (shMOLLI- *T* _1_ ) in the liver. J Magn Reson Imaging [Internet]. 2016 Jul [cited 2016 Sep 16]; Available from: http://doi.wiley.com/10.1002/jmri.25392
